# Genomic Epidemiology Reveals Multiple Introductions of Severe Acute Respiratory Syndrome Coronavirus 2 in Niigata City, Japan, Between February and May 2020

**DOI:** 10.3389/fmicb.2021.749149

**Published:** 2021-10-28

**Authors:** Keita Wagatsuma, Ryosuke Sato, Satoru Yamazaki, Masako Iwaya, Yoshiki Takahashi, Akiko Nojima, Mitsuru Oseki, Takashi Abe, Wint Wint Phyu, Tsutomu Tamura, Tsuyoshi Sekizuka, Makoto Kuroda, Haruki H. Matsumoto, Reiko Saito

**Affiliations:** ^1^Division of International Health (Public Health), Graduate School of Medical and Dental Sciences, Niigata University, Niigata, Japan; ^2^Niigata City Public Health and Sanitation Center, Niigata, Japan; ^3^Division of Health Science, Niigata City Institute of Public Health and Environment, Niigata, Japan; ^4^Division of Bioinformatics, Graduate School of Science and Technology, Niigata University, Niigata, Japan; ^5^Virology Section, Niigata Prefectural Institute of Public Health and Environmental Science, Niigata, Japan; ^6^Pathogen Genomics Center, National Institute of Infectious Diseases, Tokyo, Japan; ^7^Division of Health and Welfare, Niigata Prefectural Government Office, Niigata, Japan

**Keywords:** COVID-19, SARS-CoV-2, Japan, genomic epidemiology, active epidemiological surveillance, droplet, airborne, contact transmission

## Abstract

The coronavirus disease 2019 (COVID-19) has caused a serious disease burden and poses a tremendous public health challenge worldwide. Here, we report a comprehensive epidemiological and genomic analysis of SARS-CoV-2 from 63 patients in Niigata City, a medium-sized Japanese city, during the early phase of the pandemic, between February and May 2020. Among the 63 patients, 32 (51%) were female, with a mean (±standard deviation) age of 47.9 ± 22.3 years. Fever (65%, 41/63), malaise (51%, 32/63), and cough (35%, 22/63) were the most common clinical symptoms. The median *C*_*t*_ value after the onset of symptoms lowered within 9 days at 20.9 cycles (interquartile range, 17–26 cycles), but after 10 days, the median *C*_*t*_ value exceeded 30 cycles (*p* < 0.001). Of the 63 cases, 27 were distributed in the first epidemic wave and 33 in the second, and between the two waves, three cases from abroad were identified. The first wave was epidemiologically characterized by a single cluster related to indoor sports activity spread in closed settings, which included mixing indoors with families, relatives, and colleagues. The second wave showed more epidemiologically diversified events, with most index cases not related to each other. Almost all secondary cases were infected by droplets or aerosols from closed indoor settings, but at least two cases in the first wave were suspected to be contact infections. Results of the genomic analysis identified two possible clusters in Niigata City, the first of which was attributed to clade S (19B by Nexstrain clade) with a monophyletic group derived from the Wuhan prototype strain but that of the second wave was polyphyletic suggesting multiple introductions, and the clade was changed to GR (20B), which mainly spread in Europe in early 2020. These findings depict characteristics of SARS-CoV-2 transmission in the early stages in local community settings during February to May 2020 in Japan, and this integrated approach of epidemiological and genomic analysis may provide valuable information for public health policy decision-making for successful containment of chains of infection.

## Introduction

Since the first report of the novel severe acute respiratory syndrome coronavirus 2 (SARS-CoV-2) in Wuhan, China at the end of 2019, the coronavirus disease 2019 (COVID-19) pandemic has become an unprecedented threat to public health on a global scale (Heymann and Shindo, 2020; Wang et al., 2020). As of early June 2021, more than 170 million cases have been reported worldwide, with over 3.6 million fatalities, resulting in a significant disease burden (World Health Organization, 2021).

In Japan, the COVID-19 epidemic began on January 16, 2020, with the first confirmed case being a returnee from Wuhan, China (Furuse et al., 2020a; Muto et al., 2020). Subsequently, the number of cases increased during January and April, reaching the first peak of 720 new cases in a day on April 11. The Japanese government declared a nationwide state of emergency between April 16 and May 25, 2020, in Japan.

In the early stages of Japan’s response to the COVID-19 outbreak, the Japanese government assigned COVID-19 as a designated infectious disease within the framework of the Infectious Diseases Control Law, which requires physicians to immediately report diagnosed COVID-19 cases to the public health center in their jurisdiction, on February 01, 2020 (Ministry of Health Labour and Welfare, 2020a). The recognition of the disease as a designated infectious disease enabled the respective administrations to conduct active epidemiological surveillance and make recommendations and measures for mandatory hospitalization for quarantine purposes. Subsequently, based on the framework of the Infectious Disease Control Law, the Japanese government began implementing a cluster-based approach based on active epidemiological surveillance as part of the response to control the spread of the virus (Oshitani, 2020; Imamura et al., 2021). The aim of this strategy is to conduct intensive tracing of super-spreading events (i.e., clusters) of COVID-19 cases to investigate the activities of multiple infected individuals and to identify common sources of infection. In particular, it aims to identify cluster-borne occasions and to control transmission by restricting these occasions and encouraging behavioral changes in the general public. Preliminary epidemiological investigations by the National Task Force for COVID-19 outbreak in Japan identified hospitals and other medical facilities, as well as nursing homes and other social care facilities, as the main sources of clusters; at the individual level, many of the clusters were associated with karaoke parties, clubs, bars, and gyms, which were identified as being associated with high-risk behaviors in close proximity (Furuse et al., 2020a,b). By the end of April 2020, the Task Force reported three situations that could increase the risk of COVID-19 and started to advise the public to avoid the “three Cs”: closed spaces with poor ventilation, crowded places, and close-contact settings, which led to the initial successful containment of many chains of infection in Japan (Oshitani, 2020).

On the other hand, owing to the virus spreading from Wuhan to various countries across the world in a relatively short period of a few months, there was a need to develop a genomic analysis based on whole-genome sequencing of SARS-CoV-2 in Japan to track the transmission routes of the virus (Sekizuka et al., 2020a,b, c). This approach to genomic epidemiology has been reported in many countries, including the United States of America (United States) (Deng et al., 2020; Laiton-Donato et al., 2020), the United Kingdom (United Kingdom) (da Silva Filipe et al., 2021), China (Lu et al., 2020; Geidelberg et al., 2021), New Zealand (Geoghegan et al., 2020), Italy (Alteri et al., 2021), Scotland (da Silva Filipe et al., 2021), Austria (Popa et al., 2020), Russia (Komissarov et al., 2021), Morocco (Badaoui et al., 2021), and Brazil (Faria et al., 2021), and has proven to be a useful tool for investigating outbreaks and tracking the evolution and spread of the virus. In particular, understanding the evolution and transmission patterns of viruses after they enter new populations is important for developing effective strategies for disease prevention and control. However, to date, only a limited number of studies have evaluated the viral genome information of SARS-CoV-2 combined with epidemiological studies at a local level (prefectural-level or city-level) in Japan as a whole (Seto et al., 2021).

In this study, we report the clinical characteristics of 63 COVID-19 cases, including ribonucleic acid (RNA) viral load over the course of the disease, as well as an integrated approach of epidemiological and virological genomic data to investigate the transmission dynamics and patterns of SARS-CoV-2 in a local city, Niigata City (Niigata Prefecture, Japan), between February and May 2020. Specifically, we performed phylogenetic and phylogeographic analysis and examined the temporal changes of genotypes to identify multiple introductions of SARS-CoV-2 over time in Niigata City. These results provide insights into the use of the genomic epidemiology approach in addition to the active epidemiological surveillance for the monitoring of COVID-19 in local clusters and may provide valuable information for public health policy decision-making.

## Materials and Methods

### Study Location

Niigata City is located in the northern area of the main island on the coast of the Japan Sea and is the capital of Niigata Prefecture, Japan (Supplementary Figure 1) (Ogushi, 2021). In 2020, it had a population of approximately 800,000. Niigata City is well connected to the capital of Japan, Tokyo, with a bullet train that takes passengers to the capital in approximately 2 h.

### National Active Epidemiological Surveillance

In Japan, the national active epidemiological surveillance of COVID-19 was created based on the Infectious Diseases Control Law following the declaration of the COVID-19 as a “designated infectious disease” (National Institute of Infectious Diseases, 2020). Health authorities in 47 prefectures and 20 major cities throughout Japan take the responsibility for identifying cases, as well as putting in place various control measures to stop viral transmission within the community, including: (a) isolate patients in hospitals or hotels until qualitative reverse transcription polymerase chain reaction (RT-qPCR) test results became negative (this policy was valid from February 03 to May 29, 2020), (b) find the secondary cases and clusters by implementing active epidemiological investigation and RT-qPCR testing, and (c) identify infection sources to help prevent the spread of the virus and form clusters. The target of this active epidemiological surveillance was laboratory-confirmed cases and their close contacts.

### Definitions of Case and Contacts in Epidemiological Investigation

In March 12, 2020, a “suspected case” of COVID-19 was defined by the Ministry of Health, Welfare, and Labor in Japan as a person with common cold-like upper respiratory symptoms and/or fever ≥37.5°C, and general fatigue or breathing difficulties lasting 4 days or longer (National Institute of Infectious Diseases, 2020). For individuals in high-risk groups, such as the elderly and patients with diabetes mellitus, cardiac failure, respiratory illness, hemodialysis, immunosuppressants, or undergoing chemotherapy for cancer, the definition was adapted to those presenting symptoms and signs for 2 days or longer. Contact history to a known or suspected COVID-19 case or travel history to endemic areas before 14 days was also counted when assessing the possibility of COVID-19 infections. Laboratory-confirmed cases were defined as those with a positive test result by RT-qPCR for SARS-CoV-2 with or without the above symptoms. Close contact was defined as a person who had contact with the confirmed cases in the following conditions: (a) persons who are living or staying closely with the confirmed cases including cars or air crafts, (b) persons who consulted or took care of the confirmed cases without appropriate personal protection, (c) persons who had direct contact with contaminants, such as respiratory secretions or the body fluid of the confirmed case, and (d) persons who touched or had a conversation within the confirmed case with 2 m. Initially, tracing of close contacts was advised to start from the day of symptom onset in the confirmed case by the Ministry of Health, Welfare and Labor in Japan (National Institute of Infectious Diseases, 2020). However, in Niigata city, contact tracing was started 2 days before the onset of symptoms in confirmed cases from early March 2020 to facilitate the detection of cases of pre-symptomatic transmission. Epidemiological links were categorized into four categories: (i) epidemiological links, (ii) possible links, (iii) no epidemiological links, and (iv) imported. An “epidemiological link” was defined as a case with a close contact history, as mentioned above, with a confirmed case and for whom the infection route was identified or estimated. A “possible link” was defined as one with no close or direct contact between the patients proved, but in which a weak link existed, such as using the same facility or living in the same community. “No epidemiological link” was defined by a lack of known contact or no possible link with a confirmed case. Imported cases were defined as those with a history of travel abroad up to 1 week before the onset of illness.

### Patient Clinical Data Collection and Discharge Criteria

The Niigata City Public Health Center conducted active epidemiological surveillance based on the Infectious Diseases Control Law and collected clinical data of 63 symptomatic patients within the framework of the Nationwide Active Epidemiological Surveillance. Specifically, age, sex (male or female), presence of clinical symptoms (fever, malaise, cough, sore throat, taste and smell disturbances, runny nose, chills, loss of appetite, and diarrhea), days of hospitalization, and behavioral history (e.g., close contact or travel history) were recorded. All 63 cases identified in the present study were hospitalized for quarantine purposes under the regulations of the Infectious Diseases Control Law. The criteria for discharge of symptomatic patients in Japan from February 03 to May 29, 2020, stipulated that symptoms after admission improved, they were afebrile <37.5°C for more than 24 h, and their respiratory symptoms subsided, in addition to two negative RT-qPCR tests performed at least 24 h apart (Ministry of Health Labour and Welfare, 2020b). Therefore, multiple RT-qPCR tests were performed on a single patient at intervals of 24 h or more until two negative RT-qPCR results were confirmed.

### Collection of Clinical Specimens and Reverse Transcription Polymerase Chain Reaction Testing

A total of 247 nasopharyngeal specimens were collected from 63 symptomatic patients in Niigata City between February and May 2020, and RT-qPCR testing targeting the nucleocapsid protein of the viral genome of SARS-CoV-2 was performed at the Niigata City Institute of Public Health and Environment (Niigata City Institute of Public Health and Environment, 2020), based on laboratory protocols by the National Institute of Infectious Diseases in Tokyo, Japan (Ministry of Health Labour and Welfare, 2020b). The positive RNA samples were subjected to whole-genome sequencing. The cycle threshold (*C*_*t*_) value of an RT-qPCR run was inversely correlated with the copy number of the viral RNA. A *C*_*t*_ value higher than 40 (i.e., the limit of detection) was considered negative for SARS-CoV-2. Days from onset to sampling collection, days from onset to two negative RT-qPCR results, and the number of RT-qPCR tests per case were analyzed.

### Whole Genome Sequencing of Severe Acute Respiratory Syndrome Coronavirus 2

The whole genome sequences of SARS-CoV-2 were obtained using the PrimalSeq protocol to enrich complementary deoxyribonucleic acid (cDNA) of the SARS-CoV-2 genome by multiplex RT-qPCR amplicons using a multiplexed PCR primer set that was proposed by the Wellcome Trust ARTIC Network (2021). Two amplicons with low or zero coverage owing to the dimerization of the primers were identified. Therefore, we modified the protocol for SARS-CoV-2 genome sequencing produced by the ARTIC Network and exchanged some of the primers for multiplex PCR (Itokawa et al., 2020). Detailed information of multiplexed RT-qPCR primer sets was described in Supplementary Table 1 (Itokawa et al., 2020). The PCR products from the same clinical sample were pooled, purified, and subjected to Illumina library construction using the QIAseq FX DNA Library Kit (QIAGEN, Hilden, Germany). The NextSeq 500 or iSeq100 platform (Illumina, San Diego, CA, United States) was used to sequence the indexed libraries. Next-generation sequencing (NGS) reads were mapped to the SARS-CoV-2 Wuhan/WIV04/2019 reference genome sequence [29.9-kb single standard RNA (ss-RNA)] (GenBank ID MN908947), resulting in the specimen-specific SARS-CoV-2 genome sequence by full mapping to the reference genome. The mapped reads of the SARS-CoV-2 sequences were assembled using A5-miseq version 20140604 or SKESA version 2.3.0 to determine the full genome sequence (Coil et al., 2015). Single nucleotide variation (SNV) sites and marked heterogeneity were extracted by read mapping at a depth of ≥10× and from the region spanning nucleotides (nt) 99 to 29,796 of the Wuhan/WIV04/2019 genome sequence. The lineage or clade classification of SARS-CoV-2 was performed using those of the Global Initiative on Sharing All Influenza Data (GISAID) database^1^, PANGOLIN (version 2.0^2^ (Rambaut et al., 2020) and Nextstrain team version 8.0^3^ (Hadfield et al., 2018) (Supplementary Table 2).

### Phylogenetic and Phylogeographic Analysis and Haplotype Network

The full genome sequences of SARS-CoV-2 were downloaded from the Global Initiative on Sharing All Influenza Data (GISAID) database (see Text Footnote 1) (Shu and McCauley, 2017). For phylogenetic analysis, we used a total of 1,374 genomic sequences, including 781 global strains, 546 Japanese strains, and 47 strains from Niigata City obtained in this study. Global strains in the present study were collected from December 2019 to May 2020, which is used in the global phylogenetic tree, constructed by Nextstrain team version 8.0 (see Text Footnote 3) (Hadfield et al., 2018), and the Japanese strains from January to May 2020 excluding clade O (other) and airport quarantine isolates in Japan downloaded on December 04, 2020. As a reference sequence, Wuhan/WIV04/2019 (GISAID ID, EPI_ISL_402124), the official reference sequence from the GISAID database, was used. Multiple alignments for the full-length genome sequences of SARS-CoV-2 were performed using MAFFT version 7.475 (Katoh and Standley, 2013). The removal of spurious sequences or poorly aligned regions from a multiple sequence alignment was performed using trimAl version 1.4.rev22 (Capella-Gutiérrez et al., 2009). The maximum likelihood (ML) phylogenetic analysis was performed using IQ-TREE version 2.1.2 with Model Finder and ultrafast bootstrap test parameters (Nguyen et al., 2015; Kalyaanamoorthy et al., 2017; Hoang et al., 2018), and visualized by iTOL version 6.0 (Letunic and Bork, 2021).

Haplotype networks from genomic SNVs (HN-GSNV) for Niigata City isolates were conducted using median joining network analysis (Bandelt et al., 1999) by PopART version 1.7 (Leigh and Bryant, 2015) against the SNV created by aligning the sequences by MAFFT and excluded the gap containing sequences. SNV detection was performed using snp-sites version 2.5.1 (Page et al., 2016) after multiple alignment based on the reference sequence by MAFFT version 7.475. The annotation of the detected SNV was added by SnpEff version 5.0e (Cingolani et al., 2012).

Retrospectively trace the dispersal dynamics of SARS-CoV-2 transmission patterns in Niigata City and other global/local areas, phylogeographic analysis was performed by Nextstrain team version 8.0 (see Text Footnote 3) (Hadfield et al., 2018) against 292 global strains, 3,968 Japanese strains, and all 47 strains obtained in this present study, which had accurate collection date information. Global strains in the present study were collected from December 2019 to May 2020 and were used to construct the global phylogenetic tree using Nextstrain team version 8.0 (see Text Footnote 3) (Hadfield et al., 2018) and all Japanese strains from January to May 2020 downloaded on August 31, 2021.

### Statistical Analysis

Data were described as the mean ± standard deviation (SD) and/or median [interquartile range (IQR)] for continuous variables and frequency (%) for categorical variables. Age (years) was divided into ten categorical variables (0–9, 10–19, 20–29, 30–39, 40–49, 50–59, 60–69, 70–79, 80–89, and 90–100 years) and time from onset to sampling collection (days) into four categorical variables (≤9, 10–20, 20–30, and ≥30 days) for analysis. The Kruskal–Wallis rank-sum test was used to compare the median values of C*_*t*_* values of RT-qPCR (cycles) by the four categorical variables of time from onset to sample collection. Bonferroni correction was applied as a *post hoc* test for each pair. Spearman’s rank-order correlation coefficient (ρ) was used to investigate the association between the time from onset to sample collection and the *C*_*t*_ values of RT-qPCR. Statistical significance was set at *p* < 0.05, using a two-tailed test. All analyses were performed using EZR version 1.27 (Kanda, 2013) and STATA version 15.1 (Stata Corp., College Station, TX, United States).

### Ethical Approval and Consent to Participate

This study was conducted in accordance with the Nationwide Active Epidemiological Surveillance under the Infectious Diseases Control Law in Japan. Therefore, the approval of the ethical review board was waived, as well as the need for written consent from the patients and close contacts to collect the epidemiological and clinical data, viral sample collection, and analysis. The personal data of patients used in this study were anonymized.

## Results

### Epidemiological Dynamics

**FIGURE 1:**
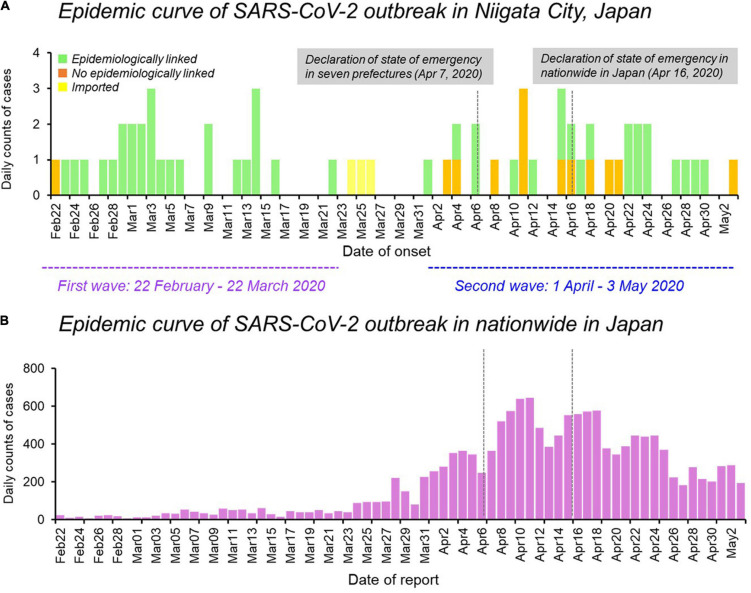
Epidemiological dynamics of SARS-CoV-2 Niigata City and nationwide in Japan. **(A)** Epidemic curve of daily cases of laboratory-confirmed SARS-CoV-2 infection in Niigata City, Japan, by symptom onset date and colored as per epidemiological linkage, between 22 February and May 03, 2020 (*n* = 63). **(B)** Epidemic curve of daily cases of laboratory-confirmed SARS-CoV-2 infection in nationwide in Japan, by reporting date and colored as purple, between 22 February and May 03, 2020 (*n* = 14,915). The purple dotted line represents the first wave by the date of onset (between 29 February and March 22, 2020) and the light blue dotted line represents the second wave by the date of onset (between 01 April and May 03, 2020). Data on the daily number of new confirmed cases in nationwide in Japan were retrieved from the website of the Ministry of Health, Labour and Welfare, Japan (https://www.mhlw.go.jp/stf/covid-19/open-data.html).

The epidemic curve of the initial 63 cases of COVID-19 in Niigata City by symptom onset date was characterized by two distinct peaks, corresponding to the first wave (between February 22 and March 22, 2020) and the second wave (between April 01 and May 03, 2020) (Figure 1). Overall, 47 (75%) of 63 patients had an epidemiological link, 13 (20%) had no apparent epidemiological links, and the remaining three (5%) were imported cases (Figure 1A and Table 1). Specifically, in the first wave, almost all cases (96%, 26/27) had epidemiological links, whereas in the second wave, less than half of the cases (36%, 12/33) had apparent epidemiological links. Between the two epidemic waves, three imported cases from Brazil, Czechia, and United States, were identified from March 24 to March 26, 2020 (Figure 1A and Table 1). The second wave of Niigata city corresponded to an increase of patients nationwide from the end of March 2020, and the first declaration of the state of emergency for seven prefectures (e.g., Tokyo and Osaka) announced on April 07, 2020 and subsequently, it was expanded to all 47 prefectures on April 16, 2020 mandating soft lockdown including Niigata (Figure 1B).

**TABLE 1:** Epidemiological characteristics of patients with COVID-19 in Niigata City, Japan between February and May 2020 (*n* = 63).

**Characteristics**	**Mean ± *SD* [Median, IQR]**	***n***	**%**
Age, years	47.9 ± 22.3 [45.0, 30.0–70.2]		
**Age categories**			
0–9 years		3	5
10–19 years		0	0
20–29 years		9	14
30–39 years		17	27
40–49 years		5	8
50–59 years		7	11
60–69 years		7	11
70–79 years		9	14
80–89 years		5	8
90–100 years		1	2

**Sex**			
Male		31	49
Female		32	51

**Clinical symptom**			
Fever (≥37.5°C at the onset symptoms)		41	65
Malaise		32	51
Cough		22	35
Sore throat		17	27
Loss of taste or smell		14	22
Runny nose		14	22
Chills		11	17
Loss of appetite		9	9
Diarrhea		2	2
Days from onset to sample collection	7.4 ± 6.5 [6.0, 6.0–9.0]		

**Epidemiologic linkage**			
Epidemiologically linked		47	75
Not epidemiologically linked		13	20
Imported		3	5

*SD, standard deviation; IQR, interquartile range. Note that P1-E is not included in this table because the patient lived in a different city, and precise clinical and virological information is not available.*

### Demographic and Clinical Characteristics

Under Japan’s Infectious Disease Control Law, all the patients were quarantined and hospitalized. Of the 63 patients who tested positive for SARS-CoV-2 by RT-qPCR between February and May 2020, 32 (51%) were female, with a mean age of 47.9 years (SD, 22.3 years), ranging from 0–9 to 90–100 years old (Table 1). All 63 patients were symptomatic, and no asymptomatic cases were found. Fever was the most common clinical symptom (41/63, 65%), followed by malaise (32 cases, 51%) and cough (22 cases, 35%). Loss of taste or smell was reported in 14 cases (22%) (Table 1). The time from onset to sample collection was 7.4 days (SD, 6.5 days). One 60-year-old patient (P5-2) developed severe pneumonia under ventilator control, however, he recovered and was discharged almost 4 months after his onset (Supplementary Table 3). No deaths occurred during the study period.

### Time-Series of Hospitalization and Negative Reverse Transcription Polymerase Chain Reaction Tests

Among the 63 cases, the mean time from onset to two negative RT-qPCR results was 19.0 days (SD, 7.98 days) (Supplementary Table 3). One case with subsequent RT-qPCR negative results after confirmation was excluded from this calculation (P5-2). In all 63 patients, the mean number of tests was 6.8 times (SD, 4.2 times) and the mean length of hospitalization was 24.2 days (SD, 15.0 days).

### Relationship Between Days Post-symptom Onset and *C*_*t*_ Value

**FIGURE 2:**
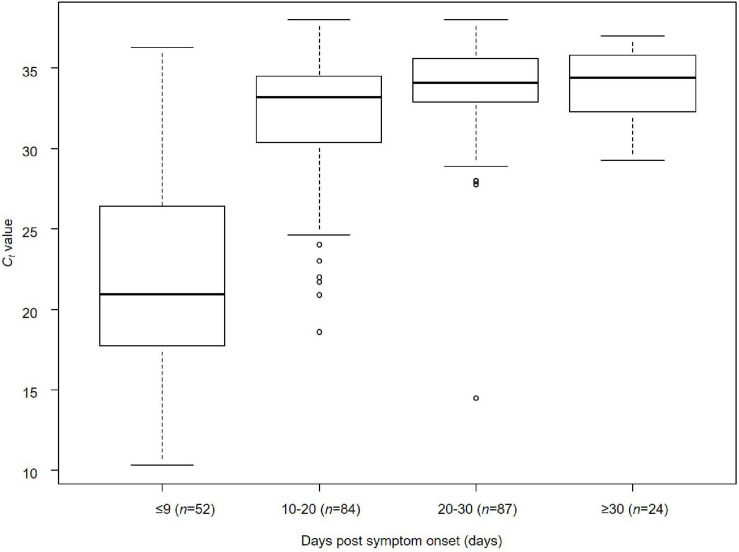
Relationship between days post-symptom onset and *C*_*t*_ value of SARS-CoV-2 in Niigata City, Japan, between February and May 2020 (*n* = 247). Kruskal–Wallis rank-sum test was used to compare the median values of *C*_*t*_ values of RT-qPCR (cycles) by the four categorical variables of time from onset to sample collection (≤9, 10–20, 20–30, and ≥30 days) (days). Bonferroni correction was applied as a *post hoc* test between each pair. A significant difference in median *C*_*t*_ values was observed between the groups ≤9 and >10 days after the onset of symptoms (Kruskal–Wallis rank-sum test, *p* < 0.001): ≤9 days vs. 10–20 days (Bonferroni correction, *p* < 0.001), ≤9 days vs. 20–30 days (Bonferroni correction, *p* < 0.001), and ≤9 days vs. ≥30 days (Bonferroni correction, *p* < 0.001). The lower and upper limits of boxes indicate the 25th and 75th percentiles, respectively; lines within boxes indicate the median values. The lines extending from the boxes indicate the range of non-outlying values. The dots represent individual points that fall outside this range (dots).

The relationship between days post-symptom onset and *C*_*t*_ values is shown for 247 samples obtained from the 63 cases by RT-qPCR (Figure 2). The median C*_*t*_* values were 20.9 cycles (IQR, 17–26 cycles; *n* = 52) for ≤9 days post-symptom onset, 33.0 cycles (IQR, 30.4–34.5 cycles; *n* = 84) for 10–20 days, 34.1 cycles (IQR, 32.9–35.7 cycles; *n* = 87) for 20–30 days, and 34.4 cycles (IQR, 32.3–35.6 cycles; *n* = 24) for ≥30 days, respectively. A significant difference in the median *C*_*t*_ values was observed between the groups ≤9 days and >10 days after the onset of symptoms (Kruskal–Wallis rank-sum test, *p* < 0.001) (Figure 2). The days post-symptom onset and *C*_*t*_ values showed a significant positive correlation (Spearman’s rank-order correlation coefficient ρ = 0.56, *p* < 0.001), suggesting an attenuation of the viral load over time. However, detailed clinical information on the relationship between viral load and severity of illness in the patients by present official surveillance framework were not available, thus, we were not able to fully assess these associations.

### Transmission Chain

**FIGURE 3:**
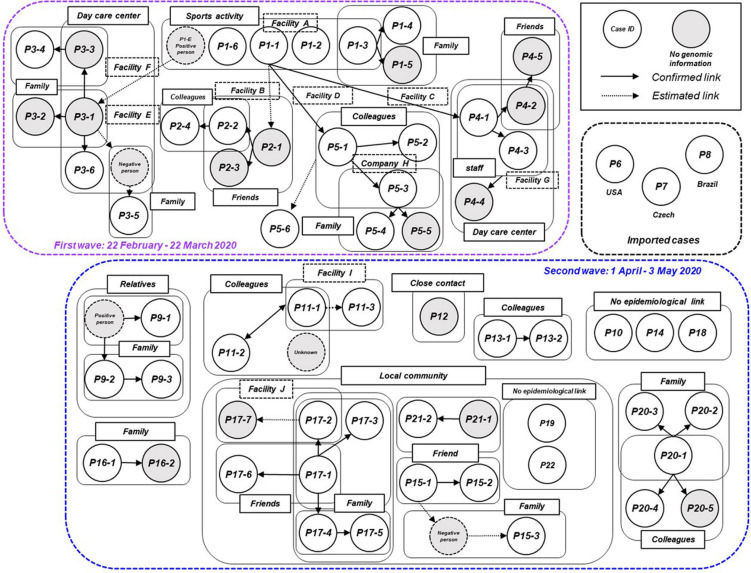
Transmission chain of the COVID-19 outbreak, Niigata City, Japan, between February and May 2020 (*n* = 63). Diagram showing the contact relationships of patients, including the 63 symptomatic cases in this study. The purple dotted box represents the first wave by the date of onset (between 22 February and March 22, 2020) and the light blue dotted box represents the second wave by the date of onset (between 01 April and May 03, 2020). The circles indicate each patient and are assigned a case ID. Gray circles indicate cases for which no genomic information was available, dotted circles indicate cases with negative qualitative reverse transcription polymerase chain reaction (RT-qPCR) results or unidentified cases. A large pale blue frame indicates common indoor environment, facility of transmission patients (e.g., family, colleagues, friends, and faculties), or community transmission. Arrows indicate established link and dotted arrows represent the most plausible link, both based on contact tracing. The case ID of each patient corresponds to the haplotype network in Figure 5 and detailed epidemiological information of Supplementary Table 4.

We plotted all 63 symptomatic patients in the first and second waves in Niigata City in 2020, and presented a transmission chain showing the relationship between the cases and the exposure histories (Figure 3 and Supplementary Table 4). Niigata City reported the first confirmed case of SARS-CoV-2 on February 29, 2020 (P1-1), almost one and a half months after the first case was reported in Japan. P1-1 was a domestically imported case, with a symptom onset of malaise and cough on 22 February, and fever on 23 February, 2020. The case had a history of travel to an endemic area outside Niigata prefecture (Tokyo metropolitan area) within 1 week before the onset to join a social gathering. It was not clear whether P1-1 had a contact with the known COVID-19 patient at the gathering. Subsequently, a cluster of four cases (P1-2, P1-3, P1-6, and P1-E) occurred indoor sports at Facility A in February 2020 (Figure 3 and Supplementary Table 4), P1-1 played the indoor sports together with the other four on February 20, 2 days before symptom onset. Thus, it was speculated as pre-symptomatic infections. From this initial cluster of cases, subsequent infections occurred in multiple secondary clusters, resulting in the first wave in Niigata City (22 February to 22 March, 2020) (Figure 1). One of the secondary infections spread from the initial patient group, namely from patient P2-1, who played a different indoor sport in the same facility (facility A) where P1-1 participated in an indoor sport activity the previous day. Although whether they shared the same locker room, athletic room, or toilet is not certain, contact infection is highly likely because they were infected despite not meeting each other and playing different sports. From P2-1, the infection spread to friends and a colleague by direct contact (P2-2, P2-3, and P2-4). P1-3 infected two family members (P1-4 and P1-5) in their household. P1-E is assumed to have infected P3-1 after both used a sports facility at the same time (facility E); however, the two patients did not see each other and played different sports, suggesting that the route of transmission may be contact infection. P1-E belongs to the first sport cluster, despite the patient living in a different city; therefore, precise clinical and virological information, including genome sequence data, was not included in this study. P3-1 spread the infection within an elderly daycare center, where P3-1 worked, to a colleague and an elderly individual (P3-3 and P3-6), then to their family members (P3-2, P3-4, and P3-5). For P1-1, the infection was spread to P4-1 and P5-1 while they participated in indoor sports activities on different days in different facilities (Facilities B and D). Case P4-1 infected two colleagues (P4-3 and P4-2) and a child (P4-4) at a children’s daycare center (Facility G), where P4-1 worked. Then, a tertiary infection occurred from P4-2 to a friend (P4-5). P5-1 caused subsequent infections in the workplace (company H) to colleagues (P5-2 and P5-3). Although direct contact with patients was not confirmed, P5-6 developed COVID-19 infection after the visit to Company H for business.

In the second wave in Niigata City, tracking the epidemiological links between patients was more difficult than in the first wave (Figure 3 and Supplementary Table 4). While several small clusters, comprised of family members and colleagues, were observed, as well as sporadic cases, the links among the clusters were difficult to confirm, unlike in the first wave. The index cases of the small clusters (P11-1, P13-1, P16-1, and P20-1) were not epidemiologically linked to each other. In addition, there was a lack of self-reported contact with persons in an endemic area (e.g., Tokyo Metropolitan area). One cluster (P9-1, P9-2, and P9-3) occurred by an extended family gathering, infected from a family member returned from another prefecture. Three cases (P10, P14, and P18) had no apparent link to the known cases or each other, while one case (P12) had a history of contact with a confirmed case in another prefecture. In addition, small clusters and sporadic cases occurred in a local area in one of the districts of Niigata City from mid-April to May. Individuals in this locality were seemingly infected through contacts that occurred during social activities, including drinking and singing karaoke with friends, neighbors, and family members (from P15-1 to -3, from P17-1 to -7, P21-1, and P21-2). However, the direct epidemiological links among these clusters in the community remain unclear. Two cases (P19 and P22) resided in the same district but had no apparent epidemiological links to others. Between the two waves, three imported cases resulting from international travels were identified (P6, P7, and P8).

### Genomic Analysis

**FIGURE 4:**
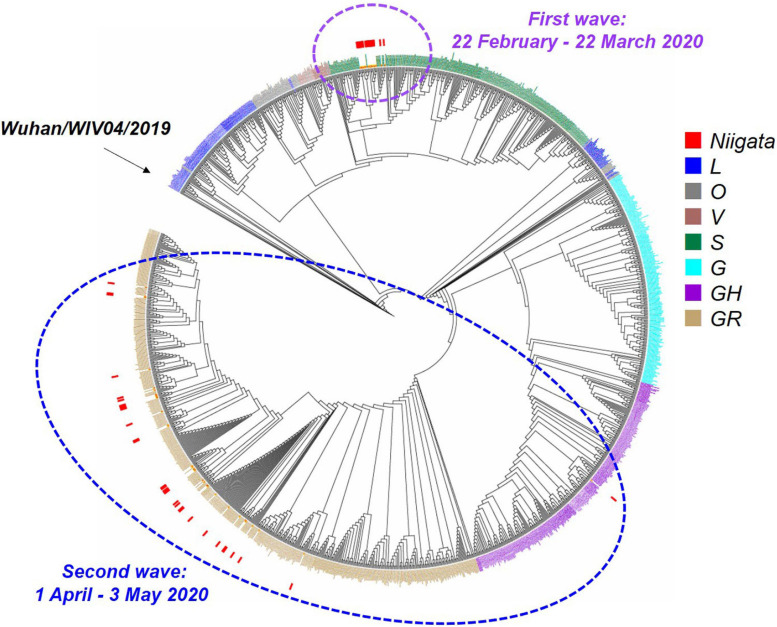
Maximum likelihood phylogenetic analysis of SARS-CoV-2 in Niigata City, Japan, between February and May 2020 (*n* = 47). This phylogenetic tree was based on the GISAID sequences of 781 global strains, 47 Niigata City strains, and 546 strains from other cities of Japan. The purple dotted circle represents the first wave by the date of onset (between 22 February and March 22, 2020) and the light blue dotted circle represents the second wave by the date of onset (between 01 April and May 03, 2020). Red squares indicate the strains detected in Niigata City, blue squares the L clade, mouse-colored squares the O clade, brown squares the V clade, dark green squares the S clade, light blue squares the G clade, dark purple squares the GH clade, and light brown squares the GR clade.

The whole viral genome was available in 47 (75%) of 63 cases. Specifically, ML phylogenetic analysis showed that the 47 isolates from Niigata City were different in the first and second wave clusters (Figure 4 and Supplementary Table 5). The first wave cluster was found to be generated by clade S, attributed to the Wuhan/WIV04/2019 haplotype, the ancestor of the global pandemic. In contrast, all isolates in the second wave cluster belonged to clade GR, the main lineage circulated in Europe, strongly suggesting that the introduction of SARS-CoV-2 in Niigata City was associated with two different SARS-CoV-2 lineages. The three imported cases belonged to GH (P6), and GR (P7 and P8), respectively.

**FIGURE 5:**
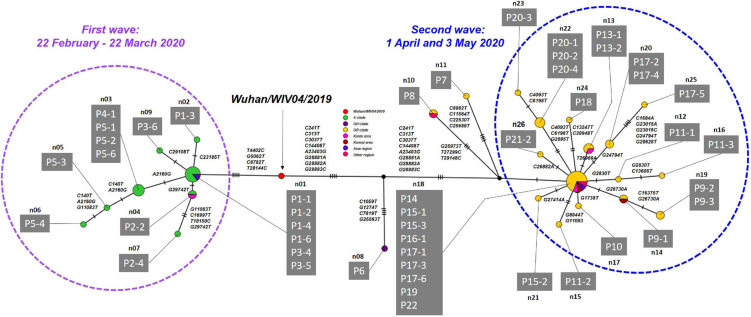
Haplotype network using genome-wide SNVs (HN-GSNVs) in Niigata City, Japan, between February and May 2020 (*n* = 47). The purple dotted circle represents the first wave by the date of onset (between 22 February and March 22, 2020) and the light blue dotted circle represents the second wave by the date of onset (between 01 April and May 03, 2020). Red circles indicate the reference sequence (Wuhan/WIV04/2019), green circles the S clade, dark purple circles the GH clade, and yellow circles the GR clade. Pink circles indicate the clusters of Kanto area prefectures isolates (Tokyo, Chiba, Kanagawa, Saitama, Gunma, and Tochigi), dark red circles the clusters of Kansai area prefectures isolate (Osaka, Kyoto, Hyogo, Shiga, Nara, and Wakayama), purple circles the clusters of Asia region isolate, and dark pink circles the clusters of other regions isolate. Haplotype clusters including each patient are represented as n01 to n26, respectively. Branches are labeled with SNVs. The case ID of each patient corresponds to the transmission chain in Figure 3 and detailed epidemiological information of Supplementary Table 4. Detailed list of nucleotide substitutions found in each haplotype constructed in Niigata City isolates compared to reference strain Wuhan/WIV04/2019 is presented in Supplementary Table 5.

Haplotype network analysis of the whole genome showed that P1-1, P1-2, and P1-6, who were exposed to infection at Facility A during the indoor sports, had identical genome sequences, while P1-3 in the same cluster had an SNV (Figure 5 and Supplementary Table 5). In the first wave, patients with secondary, tertiary, or quaternary infections (i.e., P2-2, P2-4, P3-6, P5-2, P5-3, P5-4, P3-6, and P5-6) were closely associated with two or three SNVs around the P1 index patient group (Figures 3, 5 and Supplementary Table 5). Many of the major clusters identified in the first wave had lower SNV rates than the isolates collected in the second wave, and four common nucleotide changes, T4402C, G5062T, C8782T, and T28144C, were seen from Wuhan/WIV04/2019 (Figure 5 and Supplementary Table 5), suggesting that they were closely related to the prototype SARS-CoV-2 strain. In contrast, the genome sequences of SARS-CoV-2 from the second wave possessed eight common nucleotide changes, C241T, C313T, C3037T, A14408T, A23403T, G28881A, G28882A, and G28883C from the reference, and more than 20 individual SNVs with diversified haplotype patterns (Figure 5 and Supplementary Table 5). Of note, the strains in the second wave were characterized by A23403T, Asp614Gly in spike protein that was reported to increase infectivity compared to prototype SARS-CoV-2 strain (Plante et al., 2021).

To further explore the dispersal dynamics of SARS-CoV-2 transmission patterns in Niigata City and other global/local areas, we also employed a phylogeographic inference analytic approach, using Nextstrain team version 8.0 (see Text Footnote 3) (Hadfield et al., 2018), on the whole genomes of all 47 isolates from Niigata that had accurate collection date information (Supplementary Figure 2 and Supplementary Table 5). The results indicated that the initial COVID-19 outbreak in Niigata City belonged to 19B (clade S) by Nexstrain and formed a monophyletic group that supported the epidemiological information suggesting a single introduction to the city (Supplementary Figure 2). The strains were closely related to strains in Tokyo during the same period (data not shown). In contrast, the strains in the second wave belonged to 20B (clade GR), showing polyphyletic distributions in the time-aware phylogeny by Nexstrain, and showed strong relationship with the strains mainly in Tokyo (Supplementary Figure 2). These results are indicative of the existence of multiple untraced introduction pathways in the second wave in Niigata City. Genome analysis provides useful information with which to assess where the SARS-CoV-2 strains originated from and can be used as Supplementary Information to identify and confirm epidemiological links.

## Discussion

In the present study, both epidemiological and genome-wide analyses were used to elucidate the transmission dynamics of SARS-CoV-2 in a local area of Japan during the early stages of the COVID-19 pandemic in 2020. Two waves of infection were identified in Niigata City, Japan between February and May 2020, with different patterns of infection in each wave. In the first wave, all of the cases identified were infected with the Clade S (19B) virus from China, which was prevalent in Japan in February and March (Wagatsuma et al., 2021), with the majority of these patients spreading the infection from a single index group to their family members or colleagues. Interestingly, the first wave was characterized by a local epidemic that occurred in a closed environment, namely a sports facility, which resulted in a chain of cluster-mediated infections, as described above. On the other hand, the second wave was characterized by the clade GR (20B) virus from Europe, which was prevalent in Japan since mid-March (Sekizuka et al., 2020a), with an increased number of cases with unknown routes of infection compared to the first wave. There was no commonality in age, sex, or behavior of the patients in the second wave, indicative of multiple routes of entry. Similar to our study, this type of genomic epidemiological analysis has been widely used in various local communities in other countries (Deng et al., 2020; Geoghegan et al., 2020; Laiton-Donato et al., 2020; Lu et al., 2020; Popa et al., 2020; Sekizuka et al., 2020a; Alteri et al., 2021; Badaoui et al., 2021; da Silva Filipe et al., 2021; Faria et al., 2021; Geidelberg et al., 2021; Komissarov et al., 2021). In support of the literature, our results highlight the benefits of genome surveillance to complement epidemiological surveillance and further support a comprehensive country-wide study of SARS-CoV-2 epidemiology and transmission patterns in Japan.

The most common clinical symptoms reported in the patient population included in the present study were almost identical to those of previous studies. In this study, the most common symptoms were fever (65%), followed by malaise (51%), cough (35%), and sore throat (27%). The largest Japanese registry study describing the clinical epidemiological characteristics of 2,638 hospitalized COVID-19 patients enrolled from 227 healthcare facilities in Japan as of July 07, 2020, showed similar results, with fever, cough, dyspnea, fatigue, and olfactory and gustatory disturbances in a large proportion of patients (Matsunaga et al., 2020). On the other hand, the demographic characteristics of the present study population showed a broadly uniform age distribution and an almost equal ratio of males to females. It should be emphasized that this is contradictory to the Japanese registry study, which reported a median age of 56 years (IQR, 40–71 years) for COVID-19 inpatients, with more than half of the cases comprised of male patients (58.9%, 1542/2619). The reason for this difference compared to the previous literature may be that the epidemiology in the early stages of the initial epidemic in Niigata City may not have been captured owing to the small number of cases (i.e., 63 cases). Therefore, there is a need to increase the number of cases studied in the future, as well as clarify the patient characteristics because disease severity changes over time, owing to surveillance capacity and circulating genotypes, in the case of COVID-19.

The results of the analysis of the *C*_*t*_ values of the initial 63 symptomatic individuals detected in Niigata City showed that the highest viral load was recorded after the onset of symptoms, and that the viral load decreased steadily after the onset of symptoms, with *C*_*t*_ values > 30 at 10–20 days (He et al., 2020; Singanayagam et al., 2020). Notably, our results showed a relatively large median difference of approximately 10 cycles between C*_*t*_* values ≤ 9 days and >10 days after the onset of symptoms, which is consistent with the findings of several previous studies (Arons et al., 2020; Bullard et al., 2020; Perera et al., 2020; Singanayagam et al., 2020). On the other hand, many previous studies have reported that SARS-CoV-2 is likely to be transmitted from pre-symptomatic or asymptomatic patients. Although this pre-symptomatic infection was not well known at the beginning of epidemic in Japan, we checked the contact histories of patients from 2 days prior to the onset through the information of literatures and the thorough examinations of epidemiological information of the first cluster of patients in Niigata City. P1-1 seemed to have transmitted to others in the pre-symptomatic period. The previous studies in Singapore and Tianjin City in China, where active case-finding was carried out, an estimated proportion of pre-symptomatic transmission of 48% (95% credible interval [CrI], 32–67%) and 62% (95% CrI, 50–76%), were observed, respectively (Ganyani et al., 2020). The proportion of asymptomatic infections tended to be higher in areas where active case-finding was undertaken. However, in our analysis, asymptomatic patients were not detected. It is possible that several asymptomatic infections were missed owing to the limited capacity of RT-qPCR and case tracing at the beginning of the epidemic in 2020.

The results of our epidemiological analysis in Niigata City are consistent with the results of a large epidemiological study of COVID-19 clusters previously conducted using nationwide data in Japan in the early stages of the epidemic, from January to April 2020 (Furuse et al., 2020b). Furuse et al. (2020b) found that many of the COVID-19 clusters were associated with heavy breathing in closed environments, such as singing at karaoke parties, cheering in clubs, talking in bars, and exercising in gyms, with several studies characterizing these activities as the three Cs: closed spaces, crowded places, and close-contact settings (Furuse et al., 2020a,b; Muto et al., 2020). These conditions are characterized by infection via droplet or aerosol dispersion (Jayaweera et al., 2020; Morawska and Milton, 2020; Tang et al., 2021). In the present study, we found that the initial cluster of epidemics in Niigata City between February and May 2020 was also characterized by closed environments, including indoor sports activities, between relatives and colleagues, confirming the findings of previous studies. A thorough epidemiological investigation conducted by the Niigata City Public Health Center revealed that almost all secondary infections occurred via droplet or aerosol infections. Surprisingly, at least two cases were seemingly infected by contact infections since the patients of the index cases and secondary cases did not come into direct contact. Previous studies have reported that care should be taken to avoid contact infections through common areas, such as toilets and locker rooms (Huang et al., 2020; van Doremalen et al., 2020; Groves et al., 2021). On the one hand, the Centers for Disease Control and Prevention (CDC) have recently reported that the risk of contact transmission of COVID-19 is quite low (Centers for Disease Control and Prevention, 2021). However, we would like to emphasize again that the infection risk associated with this type of transmission is not zero, and that measures, such as surface disinfection of objects, are essential, especially in areas where clusters are likely to occur, such as hospitals and sports facilities.

The results of a genealogical network study using SARS-CoV-2 genomic data in Niigata City suggested that the impact of the influx of strains from abroad may have a direct influence on local epidemics in regional cities over a short period of time. Importantly, haplotype network analysis of SARS-CoV-2 suggested that the second wave of COVID-19 in early April had a distinctly different origin from the first wave of strains. This suggests that strains that subsequently spread domestically in Japan may have entered the city via multiple routes (the haplotype network includes strains from other prefectures). Additionally, a detailed assessment of potential inter-cluster variation in SNVs showed that higher nucleotide changes were observed in the second wave than in the first wave in Niigata City further highlighting the possibility of multiple sources of importation from other regions in the second wave. Furthermore, phylogeographic inference indicates the dispersion dynamics of SARS-CoV-2 transmission patterns suggested in the first wave were monophyletic from a single source, but that of the second wave were polyphyletic with multiple sources, and Tokyo was an important central transmission hub for Niigata City. In view of the increasing number of COVID-19 cases in Europe and the detection of imported cases in Japan, the Japanese government decided to suspend the entry of European travelers into Japan on March 27, 2020 (Furuse et al., 2020a; Wagatsuma et al., 2021). Local restriction measures were only weakly imposed in Japan, and more prolonged and rigid self-restraint measures could have been more effective in mitigating the increase in COVID-19 cases until mid-March. However, owing to the lack of strict measures, an increase in the number of cases occurred in Japan after April. A recent report on the impact of local and national travel restrictions on COVID-19 in Japan strongly suggests that restrictions on domestic travel by public transport are quite effective in preventing the spread of infection (Murano et al., 2021). Given the fact that large numbers of people move at the local level, local cities are likely to be immediately affected by outbreaks in densely populated areas (e.g., Tokyo, Osaka, and Aichi). However, the rapid application of travel restrictions (especially domestic) could help delay the widespread transmission of COVID-19.

This study should be interpreted in the context of several limitations. Primarily, the present surveillance and contact-tracking data only account for the early phase of the COVID-19 epidemic in Niigata City, presenting the epidemiology of only 63 initial cases in this city. Therefore, our results may not be representative of the period of sustained local transmission following the introduction of SARS-CoV-2 into Niigata City; in addition, they do not represent the whole picture of Niigata Prefecture, which has a larger population (more than double). In future studies, the sample size must be increased to analyze the association between imported events and national and/or global pandemics through social events and variations in the viral loads. A detailed analysis is critically needed to fully assess these associations. However, because COVID-19 transmission is likely to start in populated areas (Furuse et al., 2020a; Wagatsuma et al., 2021), we believe that our study is sufficient to depict the early pictures of COVID-19 in Niigata Prefecture. Additionally, literature describing the transmission dynamics of SARS-CoV-2 at the local level (prefectural-level or city-level) during the early stages of the COVID-19 pandemic by combining epidemiological analysis and genomic information are quite limited, and the authors believe that the present study has a decent value to elaborate active surveillance by public health centers in Japan. Second, within the framework of an active nationwide epidemiological surveillance, especially in early 2020, the identification of infection contacts relies on self-reported information by index cases, which may have led to the occurrence of unconfirmed cases or routes, and a degree of incompleteness, and, therefore, bias. This may have led to an underestimation of the initial epidemiology of COVID-19 infection in Niigata City. Third, in the present study, our analysis did not consider symptomatic or asymptomatic cases. In particular, it should be noted that some cases may have been missed in our study, especially in view of previous literature, which has shown that over half of all infections caused by COVID-19 can be traced back to asymptomatic individuals (Ganyani et al., 2020; He et al., 2020; Johansson et al., 2021). Fourth, during the study period, tests were only offered to patients who met the epidemiological and clinical criteria, which was initially strict owing to the low capacity of RT-qPCR. Fifth, in the present study, only the whole viral genomes of 47 (75%) out of the 63 cases were analyzed, such that not all eligible cases were analyzed. Sixth, it was difficult to conduct a detailed analysis of the initial viral load, severity of patients’ illness, and the other potential epidemiological information associated with hospitalization and negative RT-qPCR tests. Of note, assessment of the association between these epidemiological indicators may provide useful information for better clinical impact on patient management and treatment in the early stage of emerging and reemerging infectious disease outbreaks including COVID-19 pandemic and detailed analysis is a key topic for future study. Furthermore, it is fruitful to understand the differences in patient outcomes by region and country attributable to these epidemiological factors. Finally, the relatively small sample size in the present study may have underestimated the circulation of viral strains in the surrounding areas. To explicitly characterize the dispersal dynamics of SARS-CoV-2 transmission patterns in Niigata City and other global/local areas, further phylogeographic studies are critically needed to increase the sample size. Lastly, owing to the protection of privacy, it was not possible to include detailed information, such as the date of onset and date of confirmation of individual cases or types of sports activities, owing to the need to avoid disclosing personal information.

Despite the number of limitations noted above, it should be stressed that the first 4 months of the COVID-19 outbreak in Niigata City were associated with multiple introductions associated with people returning or visiting from other prefectures in which COVID-19 was more prevalent, as well as imported cases from overseas, early community transmission, and clusters associated with large indoor events, families, and workplaces. At the same time, however, Niigata City was able to control the spread of the disease better than other prefectures in Japan in the early stages of the epidemic because of its rapid epidemiological investigations in both the first and second waves (cumulative number of COVID-19 cases in neighboring prefectures as of May 2020, approximately 200 in Toyama Prefecture and approximately 150 in Gunma Prefecture vs. approximately 70 in Niigata Prefecture) (Ministry of Health Labour and Welfare, 2020c). This led to the identification of close contacts, a 2-week stay-at-home order, and prompt testing of those with symptoms. Additionally, measures such as early restrictions on in-country movement from high-risk areas with high numbers of infected people, such as Tokyo, to regional cities would have better ensured that outbreaks did not spread in the early stages of the epidemic and that multiple clusters of ongoing community transmission were prevented. The COVID-19 epidemic in Niigata City was brought briefly under control when a state of emergency was declared in Niigata Prefecture in late April 2020. Given that Japan did not adopt a strict lockdown policy to weaken the spread of the disease, it is worth noting that restricting human movement may have been effective in preventing the spread of COVID-19. Particularly in the early stages of an emerging infectious disease outbreak, including SARS-CoV-2, protecting vulnerable local and regional health systems, and securing time for these areas to prepare for an epidemic can reduce the economic impact of a pandemic. As demonstrated in this preliminary study, combining epidemiological and genomic data is likely to provide useful insights for use in future public health intervention policies, with the possibility of applying these policies to other affected regions.

## Data Availability Statement

The datasets presented in this study can be found in online repositories. The names of the repository/repositories and accession number(s) can be found in the article/Supplementary Material.

## Ethics Statement

Ethical review and approval was not required for the study on human participants in accordance with the local legislation and institutional requirements. Written informed consent from the participants’ legal guardian/next of kin was not required to participate in this study in accordance with the national legislation and the institutional requirements.

## Author Contributions

KW, RyS, SY, MI, YT, AN, and ReS designed the study. RyS, SY, MI, YT, AN, and TT collected the data. KW, RyS, TS, MK, TA, WWP, and ReS analyzed the data. KW drafted the manuscript. ReS supervised the study. RyS, SY, MI, YT, AN, MO, TS, MK, TA, TT, and HM made critical revisions to the manuscript for important intellectual content and gave final approval of the manuscript. All authors took responsibility for the integrity of the data and the accuracy of the data analysis. The opinions, results, and conclusions reported in this manuscript are those of the authors and are independent of the funding bodies.

## Conflict of Interest

The authors declare that the research was conducted in the absence of any commercial or financial relationships that could be construed as a potential conflict of interest.

## Publisher’s Note

All claims expressed in this article are solely those of the authors and do not necessarily represent those of their affiliated organizations, or those of the publisher, the editors and the reviewers. Any product that may be evaluated in this article, or claim that may be made by its manufacturer, is not guaranteed or endorsed by the publisher.

## Footnotes

1
https://www.gisaid.org/

2
https://github.com/cov-lineages/pangolin

3
https://nextstrain.org/ncov/global
