# Supplementary material for: Genomic Epidemiology Reveals Multiple Introductions of Severe Acute Respiratory Syndrome Coronavirus 2 in Niigata City, Japan, Between February and May 2020
Source: Front Microbiol. 2021 Oct 28;12:749149. doi: 10.3389/fmicb.2021.749149 (PMC8581661; doi:10.3389/fmicb.2021.749149)
Supplement: Supplementary file 1 [file Data_Sheet_1.docx]

Supplementary Material

## Supplementary Figures


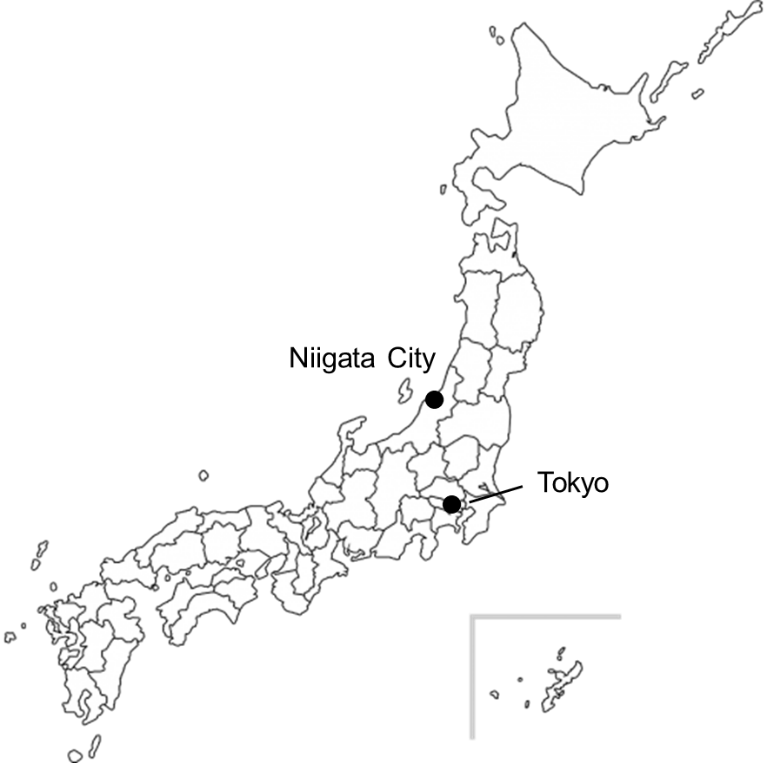


**Supplementary Figure 1. Geographical location of the study in Japan.** Niigata City (Niigata Prefecture, Japan) was chosen as the study area. Niigata City has good access to the capital of Japan, Tokyo, with passengers arriving in approximately two hours by bullet train.


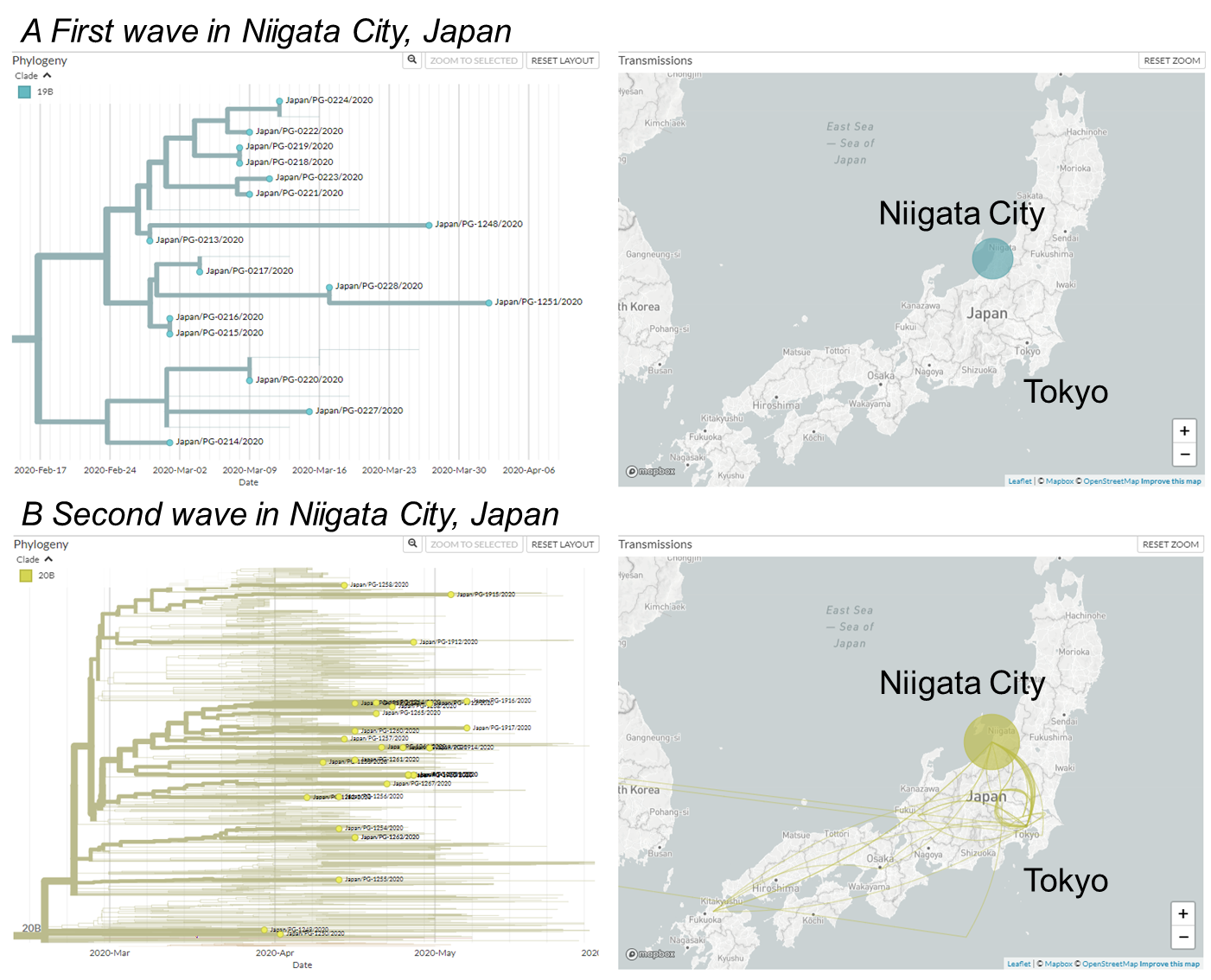


**Supplementary Figure 2. Phylogeographic analysis of SARS-CoV-2 identified in Niigata City, Japan and other global/local areas between February and May 2020.** (A) First wave (between February 29 and March 22, 2020) of SARS-CoV-2 outbreak in Niigata City, Japan. (B) Second wave (between April 1 and May 3, 2020) of SARS-CoV-2 outbreak in Niigata City, Japan. The left figure shows the time-aware phylogenetic reconstruction of SARS-CoV-2 isolates in Niigata City and other global/local areas and the right figure represents the phylogeographic of migration patterns, respectively. Phylogeographic analysis was conducted by Nextstrain team version 8.0 (<https://nextstrain.org/ncov/global>).

## Supplementary Tables

**Supplementary Table 1. List of primers for multiplex RT-qPCR in whole genome sequencing of SARS-CoV-2.**

**Supplementary Table 2. Global Initiative on Sharing All Influenza Data (GISAID) accession IDs of SARS-CoV-2 cases in Niigata City, Japan, between February and May 2020 (*n* = 63).** Sequences were not registered in the GISAID database because of multiple gaps for P1-5, P2-1, P2-3, P3-1, P3-2, P3-3, P4-2, P4-3, P4-4, P4-5, P5-5, P12, P16-2, P17-7, P20-5, and P21-1. Summary of next-generation sequencing (NGS) reads were included.

**Supplementary Table 3. Summary of clinical course and RT-qPCR testing for SARS-CoV-2 in Niigata City, Japan, between February and May 2020 (*n* = 63).** Black circles indicate days when quantitative reverse transcription PCR (RT-qPCR) was positive and white circles indicate days when RT-qPCR was negative. Orange indicates the length of hospital stay (days). The case ID of each patient corresponds to the transmission chain in Figure 3 and the haplotype network in Figure 5. Note that P1-E is not included in this table because the patient lived in a different city, and precise clinical and virological information is not available.

**Supplementary Table 4. Epidemiological information of COVID-19 cases in Niigata City, Japan, between February and May 2020 (*n* = 63).**

| **Number** | **Case ID** | **Epidemiological information** |
| --- | --- | --- |
| 1 | P1-1 | Visited Tokyo within one week before the onset. A visitor at Facility A, Facility B, Facility C, and Facility D. |
| 2 | P1-2 | A visitor at Facility A. Contacted with P1-1, P1-3, and P1-6. |
| 3 | P1-3 | A visitor at Facility A. Contacted with P1-1, P1-2, and P1-6. |
| 4 | P1-4 | A family relationship with P1-3 and P1-5. |
| 5 | P1-5 | A family relationship with P1-3 and P1-4. |
| 6 | P1-6 | A visitor at Facility A. Contacted with P1-1, P1-2, and P1-3. |
| 7 | P2-1 | A visitor at Facility B. |
| 8 | P2-2 | P2-1 and P2-3 are friends. |
| 9 | P2-3 | P2-1 and P2-2 are friends. |
| 10 | P2-4 | Colleague of P2-2. |
| 11 | P3-1 | A visitor at Facility E (Facility E was visited by positive person who visited Facility A (P1-E)). Staff of Facility F. |
| 12 | P3-2 | A family relationship with P3-1. |
| 13 | P3-3 | A visitor at Facility F. Contacted with P3-1. |
| 14 | P3-4 | A family relationship with P3-3. |
| 15 | P3-5 | Family member of Facility F user who tested negative. |
| 16 | P3-6 | A visitor at Facility F. Contacted with P3-1. |
| 17 | P4-1 | A visitor at Facility C. Contacted with P1-1. Staff of Facility G. |
| 18 | P4-2 | Colleagues of P4-1 and P4-3. Staff of Facility G. |
| 19 | P4-3 | Colleagues of P4-1 and P4-2. Staff of Facility G. |
| 20 | P4-4 | A visitor at Facility G. |
| 21 | P4-5 | P4-2 is friend. |
| 22 | P5-1 | A visitor at Facility D. Contacted with P1-1. Staff of company H. |
| 23 | P5-2 | Colleagues of P5-1 and P5-3. Staff of company H. |
| 24 | P5-3 | Colleague of P5-1 and P5-2. Staff of company H. |
| 25 | P5-4 | P5-3 and P5-5 are family. |
| 26 | P5-5 | P5-3 and P5-4 are family. |
| 27 | P5-6 | A visitor at company H. |
| 28 | P6 | Returnees from abroad. |
| 29 | P7 | Returnees from abroad. |
| 30 | P8 | Returnees from abroad. |
| 31 | P9-1 | Relatives of P9-1 and P9-3. |
| 32 | P9-2 | Relative of P9-1. P9-3 is family. |
| 33 | P9-3 | Relative of P9-1. P9-2 is family. |
| 34 | P10 | Unknown infection route. |
| 35 | P11-1 | Unknown infection route. A visitor at Facility I. |
| 36 | P11-2 | Colleague of P11-1. |
| 37 | P11-3 | A visitor at Facility I. |
| 38 | P12 | Contacted with positive person in other areas. |
| 39 | P13-1 | Unknown infection route. |
| 40 | P13-2 | Colleague of P13-1. |
| 41 | P14 | Unknown infection route. |
| 42 | P15-1 | Unknown infection route. |
| 43 | P15-2 | P15-1 is friend. |
| 44 | P15-3 | Family members of acquaintance of P15-1. |
| 45 | P16-1 | Unknown infection route. |
| 46 | P16-2 | Family member of P16-1. |
| 47 | P17-1 | Unknown infection route. |
| 48 | P17-2 | Family member of P17-1, P17-3, P17-4, and P17-5. A visitor of Facility J. |
| 49 | P17-3 | Family member of P17-1, P17-2, P17-4, and P17-5. |
| 50 | P17-4 | Family member of P17-1, P17-2, P17-3, and P17-5. |
| 51 | P17-5 | Family member of P17-1, P17-2, P17-3, and P17-4. |
| 52 | P17-6 | P17-1 is friend. |
| 53 | P17-7 | A visitor of Facility J. |
| 54 | P18 | Unknown infection route. |
| 55 | P19 | Unknown infection route. |
| 56 | P20-1 | Unknown infection route. |
| 57 | P20-2 | Family member of P20-2 and P20-3. |
| 58 | P20-3 | Family member of P20-1 and P20-2. |
| 59 | P20-4 | Colleagues of P20-1 and P20-5. |
| 60 | P20-5 | Colleagues of P20-1 and P20-4. |
| 61 | P21-1 | Unknown infection route. |
| 62 | P21-2 | Family member of P21-1. |
| 63 | P22 | Unknown infection route. |

The case ID of each patient corresponds to the transmission chain in Figure 3 and the haplotype network in Figure 5. Note that P1-E is not included in this table because the patient lived in a different city, and precise clinical and virological information is not available.

**Supplementary Table 5. List of nucleotide substitutions found in each haplotype constructed in Niigata City isolates compared to reference strain Wuhan/WIV04/2019.** The period indicates the same nucleotide as the reference sequence.
